# Real-world effectiveness of sofosbuvir/velpatasvir, glecaprevir/pibrentasvir, and sofosbuvir/velpatasvir/voxilaprevir against genotype 3 hepatitis C virus infection: a systematic review and meta-analysis

**DOI:** 10.3389/fgstr.2025.1511150

**Published:** 2025-02-12

**Authors:** Liwei Zhuang, Junnan Li, Yu Zhang, Shibo Ji, Huichun Xing

**Affiliations:** ^1^ Center of Liver Disease Division 3, Beijing Ditan Hospital, Capital Medical University, Beijing, China; ^2^ Medical Research Group, Peking University Ditan Teaching Hospital, Beijing, China; ^3^ Department of Science and Education, Beijing Ditan Hospital, Capital Medical University, Beijing, China

**Keywords:** real-world effectiveness, sofosbuvir/velpatasvir, glecaprevir/pibrentasvir, sofosbuvir/velpatasvir/voxilaprevir, genotype 3 HCV

## Abstract

**Introduction:**

Direct antiviral agents (DAAs) have dramatically changed the landscape of liver diseases associated with chronic hepatitis C virus (HCV) infection. However, limited data are available on the antiviral effect of sofosbuvir (SOF) + velpatasvir (VEL) ± ribavirin (RBV), SOF + VEL + voxilaprevir (VOX), and glecaprevir (GLE) + pibrentasvir (PIB) in treating patients infected with HCV GT3 in a real-world setting.

**Methods:**

Using the EMBASE, PubMed, and Cochrane Library databases, articles were screened from 1 January 2016 to 1 June 2024. The sustained virologic response (SVR) rates were analyzed using the Freeman–Tukey double arcsine transformation in a random-effects model in R4.1.0 software.

**Results:**

We recruited 3,177 patients with HCV GT3 in 19 studies from 9 countries. The pooled SVR12/24 rate of the three evaluated regimens was 94.00% (95% CI: 90.87-96.59%). Furthermore, the SVR rate was 83.81% (95% CI: 75.70-90.62%) in patients receiving SOF+VEL+VOX; 94.98% (95% CI: 92.02-97.33%) in patients receiving SOF+VEL ± RBV; and 96.96% (95% CI: 93.20-99.45%) in patients receiving GLE+PIB. The pooled SVR12/24 rate of the three regimens was 95.70% (95% CI: 91.74-98.58%) and 90.50% (95% CI: 83.50-95.90%) in non-cirrhotic and cirrhotic patients, respectively. The pooled SVR rate was 96.79% (95% CI: 93.37-99.13%) and 88.41% (95% CI: 82.67-93.22%) in treatment-naive and treatment-experienced patients, respectively.

**Conclusion:**

SOF+VEL ± RBV, GLE+PIB, and SOF+VEL+VOX had good antiviral effectiveness for chronic HCV-GT3 infection in real-world settings. Factors such as cirrhosis and treatment experience, especially previous DAA treatment failure, may influence the SVR rate.

## Introduction

Approximately 58 million individuals have been infected by the hepatitis C virus (HCV) in the world and 290,000 patients died from diseases associated with HCV in 2019 (1). A worldwide health sector strategy to eliminate HCV by 2030 was proposed by the World Health Organization(WHO)6 years ago (2). The sustained virologic response (SVR) has been improved significantly with the clinical application of direct-acting antivirals (DAAs) in recent years (3–9). It is reported that SVR rates of different DAAs are variable depending on the HCV genotype (GT), especially genotype 3 (10–12). Significant progress in the inhibition of HCV replication has been achieved by using new drug regimens and drug combinations such as sofosbuvir (SOF) + velpatasvir (VEL) ± ribavirin (RBV) in 2016 and SOF + VEL + voxilaprevir (VOX) and glecaprevir (GLE) + pibrentasvir (PIB) in 2017, which were approved by the European Medicine Agency (EMA) or the United States Food and Drug Administration (FDA) (13).

The antiviral effectiveness of DAAs may be decreased in a real-world setting because of poor compliance and the population diversity of patients (14–17). There is a dearth of analysis on the antiviral effectiveness of SOF+VEL ± RBV, SOF+VEL+VOX, and GLE+PIB in a real-world setting.

Thus, to evaluate the pooled SVR rate against HCV-GT3 infection in a real-world setting, we systematically searched and analyzed the latest data on SOF+VEL ± RBV, SOF+VEL+VOX, and GLE+PIB.

## Methods

### Literature search method

Using EMBASE, PubMed, and Cochrane Library, studies were searched for from 1 January 2016 to 1 June 2024 using the following terms: (“Epclusa” OR “velpatasvir” AND “sofosbuvir”) OR (“Mavyret” OR “pibrentasvir” AND “glecaprevir”) OR (“Vosevi” OR “voxilaprevir” AND “velpatasvir” AND “sofosbuvir”).

### Inclusion and exclusion criteria

Two independent researchers screened the abstracts and titles of potentially eligible publications. A full-text review of the selected articles was then performed for advanced selection in accordance with the criteria for inclusion and exclusion. Objections were discussed and resolved with a third party. The criteria for inclusion were: subject (patients infected with HCV GT3 chronically); intervention (SOF+VEL± RBV, GLE+PIB, or SOF+VEL+VOX); primary outcome (SVR rate after 8-24 weeks); and study design (real-world study). The following exclusion criteria were used: A) inaccessibility of valid data on HCV-GT3; B) assessing fewer than 10 cases; and C) meta-analyses, summaries, or case reports.

### Data extraction

Two authors independently extracted the data. Data on the demographics, SVR12, therapy duration, average HCV RNA concentration at baseline, drug dosage, treatment regimen, and virological failure were extracted using standardized forms from the articles.

### Data analysis

The SVR rates were analyzed using the Freeman–Tukey double arcsine transformation in a random-effects model. Furthermore, Egger’s test assessed publication bias, and the data analysis was conducted using R4.4.1 software. The output results do not involve ethical issues, and the research was exempted from ethical review by the Ethics Committee.

## Results

### Main characteristics of the populations and studies

**Figure 1:**
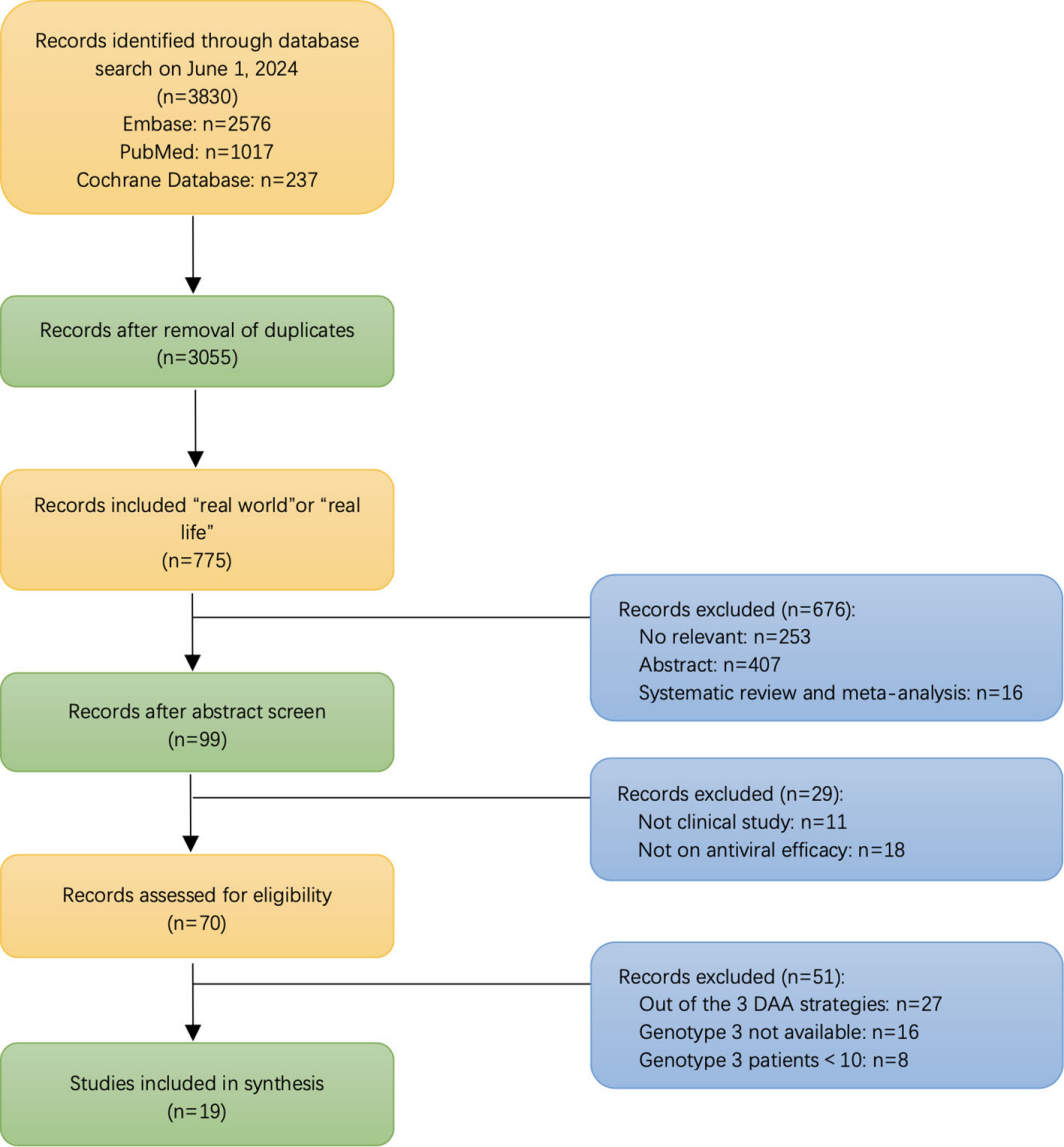
A flowchart depicting the article selection process.

**Figure 2:**
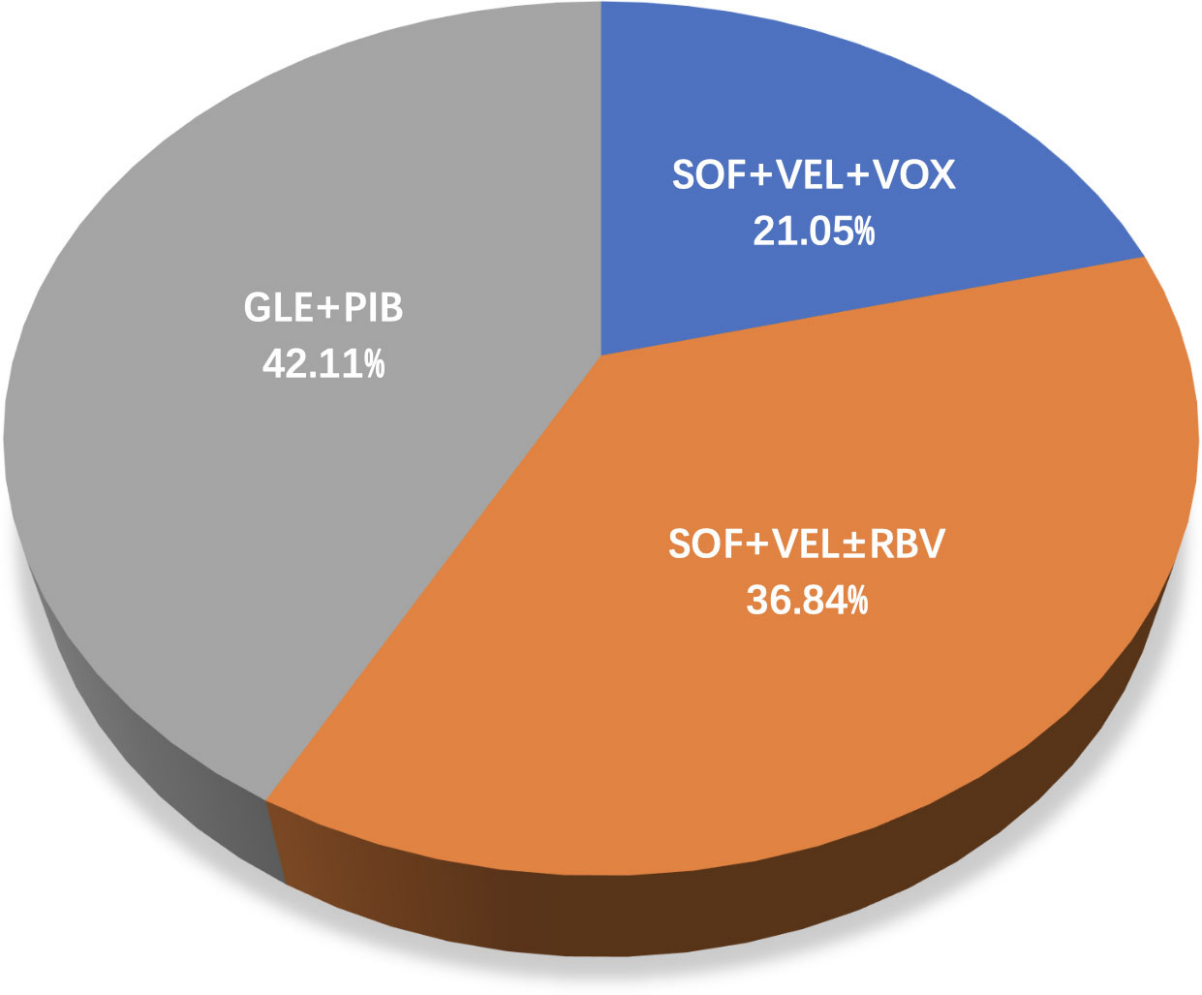
A pie chart showing the distribution of three regimens.

Overall, 3,177 HCV GT3-infected individuals were recruited in the 19 included articles, selected from a total of 3443 articles step by step as shown in **Figure 1**. These studies were conducted in eight countries: Italy (n=4), the USA (n=4), China (n=3), Germany (n=2), Japan (n=2), Denmark (n=1), Myanmar (n=1), Spain (n=1), and the UK(n=1). Of the studies, 42.11% were on GLE+PIB (8/19), 36.84% on SOF+VEL ± RBV (7/19), and 21.05% on SOF+VEL+VOX (4/19), as presented in **Figure 2**.

The patients’ clinical and demographic characteristics are shown in **Table 1**. The stages of liver disease in the patients infected by genotype 3 HCV included in these real-world studies ranged from hepatitis, advanced fibrosis, and compensated cirrhosis to decompensated cirrhosis. Some patients had at least one of the following refractory comorbidities: HIV/HBV coinfection, hepatocellular carcinoma (HCC), diabetes, history of liver transplantation, renal failure, alcohol abuse, intravenous drug use, and history of previous DAA treatment failure.

**Table 1:** The demographic characteristics of the patients.

Author, Year	Region	Sample size	Pre-treatment HCV RNA (log10 IU/mL)	Age (years)	Male, No. (%)	Demographic characteristics	Regimen, duration (weeks)	SVR12/24, No.	Virological failure^*^
Mangia et al., 2019 (18)	Italy	205	2.53 ± 4.3	52.9	175 (85.3)	72.8% treatment naïve; 54.1% transient elastography results >20KPa; 18.5% alcohol abuse; 17.6% with diabetes; 14.6% past intravenous drug use; 9.8% HIV positive; 5.3% with HCC; all with cirrhosis.	SOF+VEL, 12	200	5
Llaneras et al., 2019 (19)	Spain	30	NA	NA	NA	43.3% with cirrhosis; all treated with DCV and SOF or LDV or VEL.	SOF+VEL+VOX, 12	24	6
Hlaing et al., 2019 (20)	Myanmar	83	NA	NA	NA	67.6% with cirrhosis or advanced fibrosis; 6.16% treatment-experienced: Peg-IFN or DAA-based.	SOF+VEL ± RBV, 12-24	75	8
Degasperi et al., 2019 (21)	Italy	42	NA	NA	NA	All failed with OBV/PTV-r + DSV, or SOF and LDV or VEL	SOF+VEL+VOX ± RBV, 12	33	3
D**’**Ambrosio et al., 2019 (22)	Italy	68	NA	NA	NA	NA	GLE+PIB, 8-16	66	2
Berg et al., 2019 (23)	Germany	176	NA	NA	NA	Non-cirrhotic and treatment-naïve patients treated for 8 weeks, 12 were cirrhotic and treatment-naïve, 16 were treatment-experienced.	GLE+PIB, 8-16	174	NA
Belperio et al., 2019 (24)	USA	1735	6.05	58.95	1662 (95.8)	26.5% with cirrhosis; 9.7% treatment experienced: SOF/LDV ± RBV, PegIFN+RBV, SOF+ PegIFN+RBV, SOF+RBV;2.42% with HCC.	SOF+VEL ± RBV, 12-24	1573	NA
Belperio et al., 2019 (25)	USA	45	6.1	59.7	NA	51.1% with cirrhosis; 37.8% with diabetes; 4.44% with HCC; 2.22% with HIV coinfected; all treated with DCV and SOF or LDV or VEL.	SOF+VEL+VOX, 12	42	NA
Felden et al., 2018 (26)	Germany	222	NA	NA	NA	26.6% with cirrhosis; 24.8% treatment-experienced: Peg-IFN ± RBV or DAA-based; 8.56% with HIV coinfected.	SOF+VEL ± RBV, 12	213	NA
Tao et al., 2018 (27)	China	21	6.04	37.38	13 (59.3)	23.8% with cirrhosis.	SOF+VEL, 12-24	21	0
Mangia et al., 2019 (28)	Italy	204	NA	NA	NA	57.4% with F3-F4 stage; 43.14% PWID; 42.6% with F0-F2; 12.25% with ribavirin.	SOF+VEL ± RBV, 12	198	NA
Toyoda et al., 2020 (29)	Japan	14	6.5	46	6 (42.9)	42.9% with cirrhosis; 28.6% with HCC; 21.4% DAA-experienced: DAA-based.	GLE+PIB, 12	13	1
Nozaki et al., 2020 (30)	Japan	20	NA	54	9 (45.0)	75.0% treatment-naïve; 30.0% with cirrhosis; 30.0% with HCC; 25.0% treated by SOF/RBV or DCV/ASV.	GLE+PIB, 12	16	4
Smith et al., 2021 (31)	UK	62	6.4	57	53 (85.5)	59.67% with cirrhosis; 14.5% with prior liver transplant;12.9% with HCC; all with previous DAA treatment failure.	SOF+VEL+VOX, 8-24	50	NA
Chen et al., 2021 (32)	China Taiwan	86	NA	NA	NA	87.21% treatment-naïve; 79.07% without cirrhosis;20.93% with cirrhosis; 12.79% treatment-experienced.	GLE+PIB, 8-16	82	NA
Curry et al., 2021 (33)	USA	57	NA	43	22 (38.6)	23% CKD Stage 1-3; 5% CKD Stage 4-5;all treatment-naïve and non-cirrhotic.	GLE+PIB, 8	57	0
Solomon et al., 2022 (34)	USA	80	NA	43	NA	All treatment-naïve.	SOF+VEL, 12	73	NA
Madsen et al., 2022 (35)	Denmark	11	NA	NA	NA	All treatment-naïve and without significant liver fibrosis.	GLE+PIB, 4-8	10	NA
Chang et al., 2021 (36)	China Taiwan	11	NA	NA	NA	All treatment-naïve.	GLE+PIB, 8	11	NA

^*^ denotes a relapse or breakthrough or undefined virological failure.

### Pooled SVR rate for all cases

**Figure 3:**
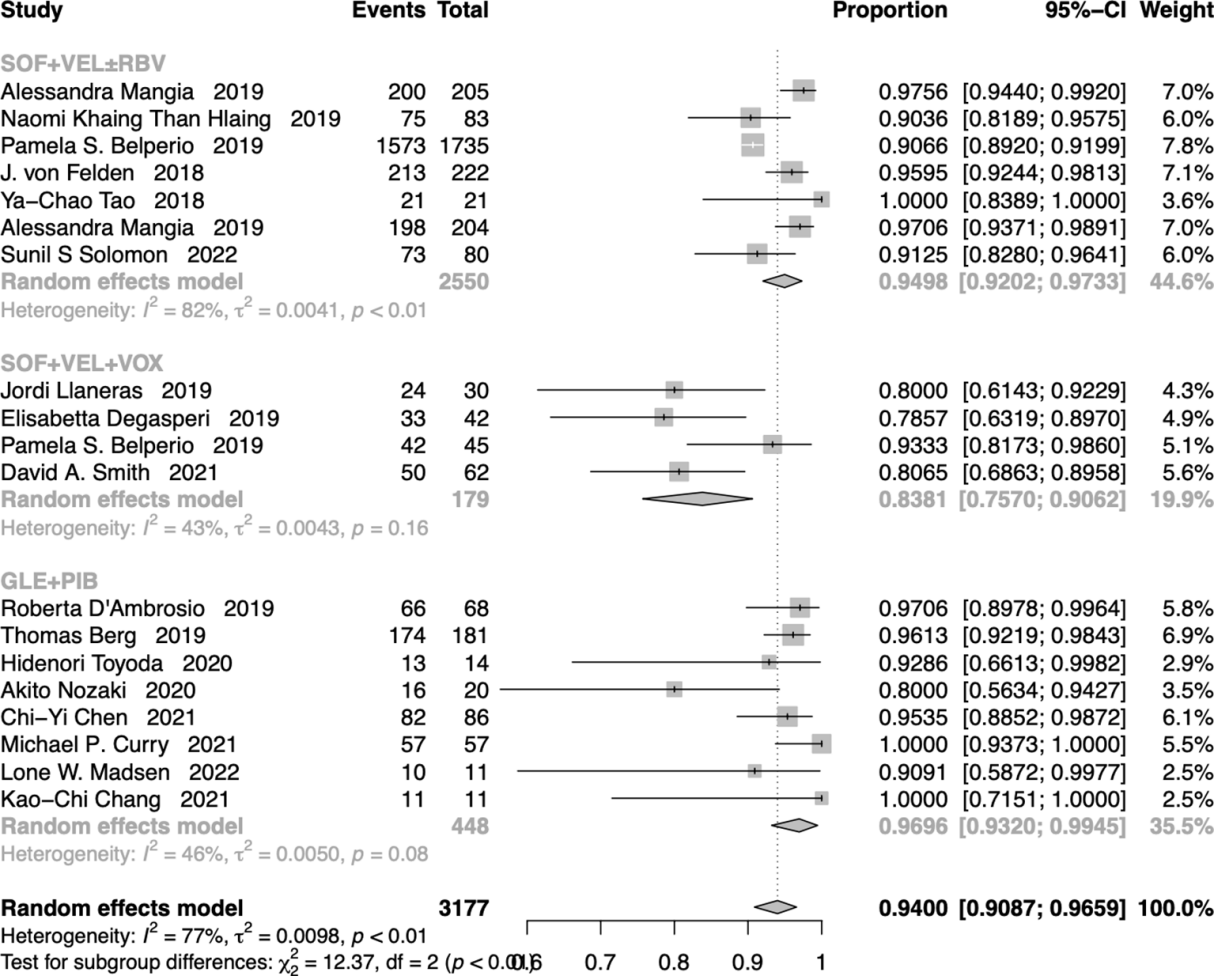
The forest plots of the pooled SVR rates for all patients.

The pooled SVR12/24 rate for cases that received SOF+VEL+VOX, SOF+VEL ± RBV, and GLE+PIB was 94.00% (95% CI: 90.87-96.59%) (**Figure 3**). In addition, the SVR12/24 rate was 83.81% (95% CI: 75.70-90.62%) in cases that received SOF+VEL+VOX, 94.98% (95% CI: 92.02-97.33%) in cases that received SOF+VEL ± RBV, and 96.96% (95% CI: 93.20-99.45%) in cases that received GLE+PIB.

### Stratification assessment of non-cirrhotic cases

**Figure 4:**
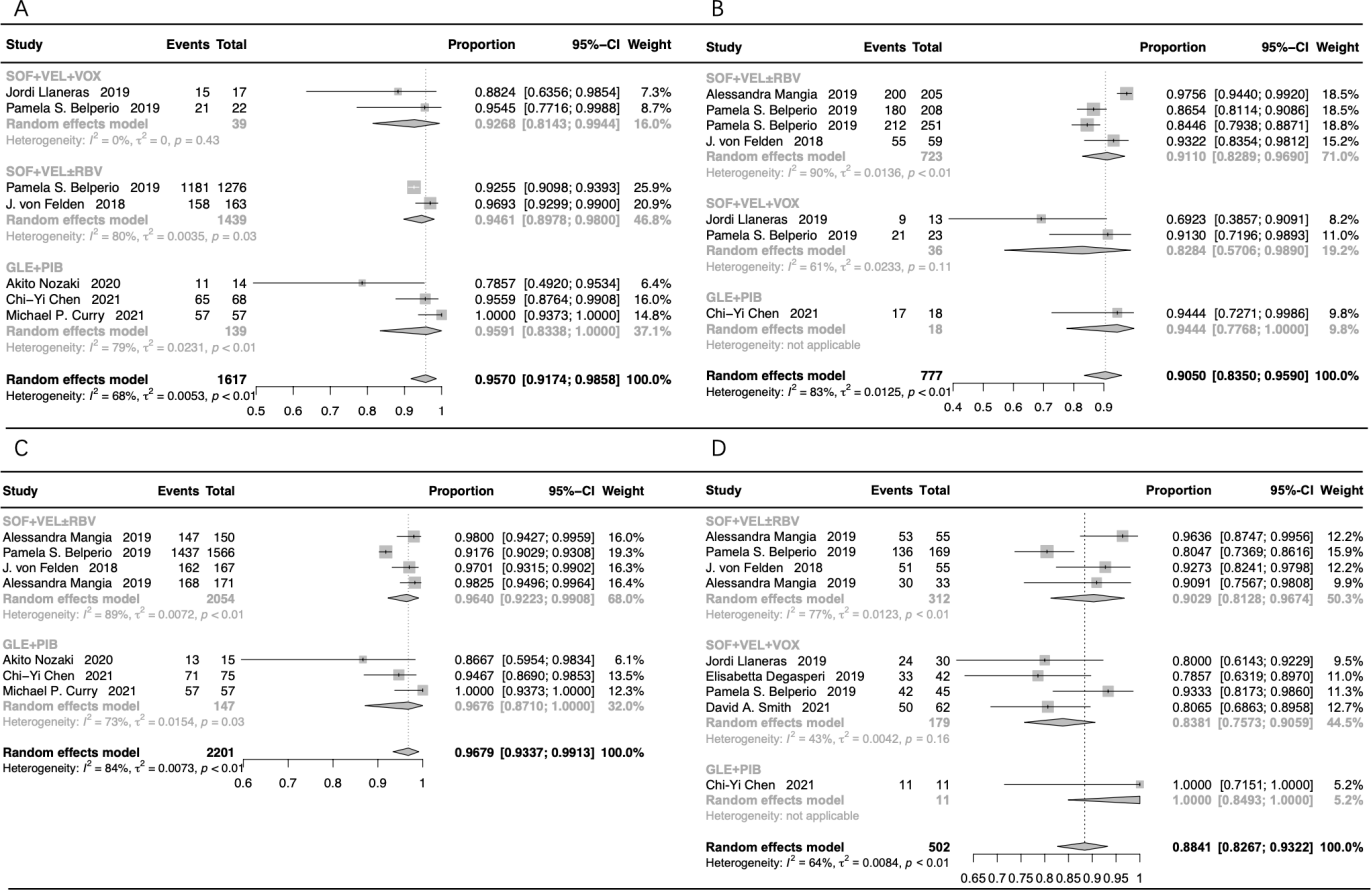
The forest plots of the stratification analysis. **(A)** The forest plots of SVR rates in non-cirrhotic patients; **(B)** the forest plots of SVR rates in cirrhotic patients; **(C)** the forest plots of SVR rates in treatment-naive patients; **(D)** the forest plots of SVR rates in treatment-experienced patients.

The subgroup assessment of non-cirrhotic cases showed that the pooled SVR12/24 rate was 95.70% (95% CI: 91.74-98.58%) in cases that received GLE+PIB, SOF+VEL+VOX, and SOF+VEL ± RBV (**Figure 4A**). Moreover, the SVR12/24 rate was 95.91% (95% CI: 83.38-100%) in cases that received GLE+PIB, 92.68% (95% CI: 81.43-99.44%) in cases that received SOF+VEL+VOX, and 94.61% (95% CI: 89.78-98.00%) in cases that received SOF+VEL ± RBV.

### Stratification assessment of cirrhotic cases

The pooled SVR12/24 rate was 90.50% (95% CI: 83.50-95.90%) in cirrhotic patients treated with GLE+PIB, SOF+VEL+VOX, and SOF+VEL ± RBV (**Figure 4B**). The SVR12/24 rate was 91.10% (95% CI: 82.89-96.90%) in cases that received SOF+VEL ± RBV, 94.44% (95% CI: 77.68-100%) in cases that received GLE+PIB, and 82.84% (95% CI: 57.06-98.90%) in cases that received SOF+VEL+VOX.

### Stratification assessment of treatment-naive cases

The subgroup assessment of the treatment-naive cases showed that the pooled SVR12/24 rate in cases that received GLE+PIB and SOF+VEL ± RBV was 96.79% (95% CI: 93.37-99.13%), as presented in **Figure 4C**. In addition, the SVR rate in cases that received GLE+PIB and SOF+VEL ± RBV was 96.76% (95% CI: 87.10-100%) and 96.40% (95% CI: 92.23-99.08%), respectively.

### Stratification assessment of treatment-experienced cases

The pooled SVR12/24 rate for the treatment-experienced cases with HCV-GT3 infection that received SOF+VEL+VOX, SOF+VEL ± RBV, and GLE+PIB was 88.41% (95% CI: 82.67-93.22%), as presented in **Figure 4D**. Furthermore, the corresponding SVR12/24 rate was 83.81% (95% CI: 75.73-90.59%), 90.29% (95% CI: 81.28-96.74%), and 100% (95% CI: 84.93-100%), respectively.

### Risk of bias and quality assessment

**Figure 5:**
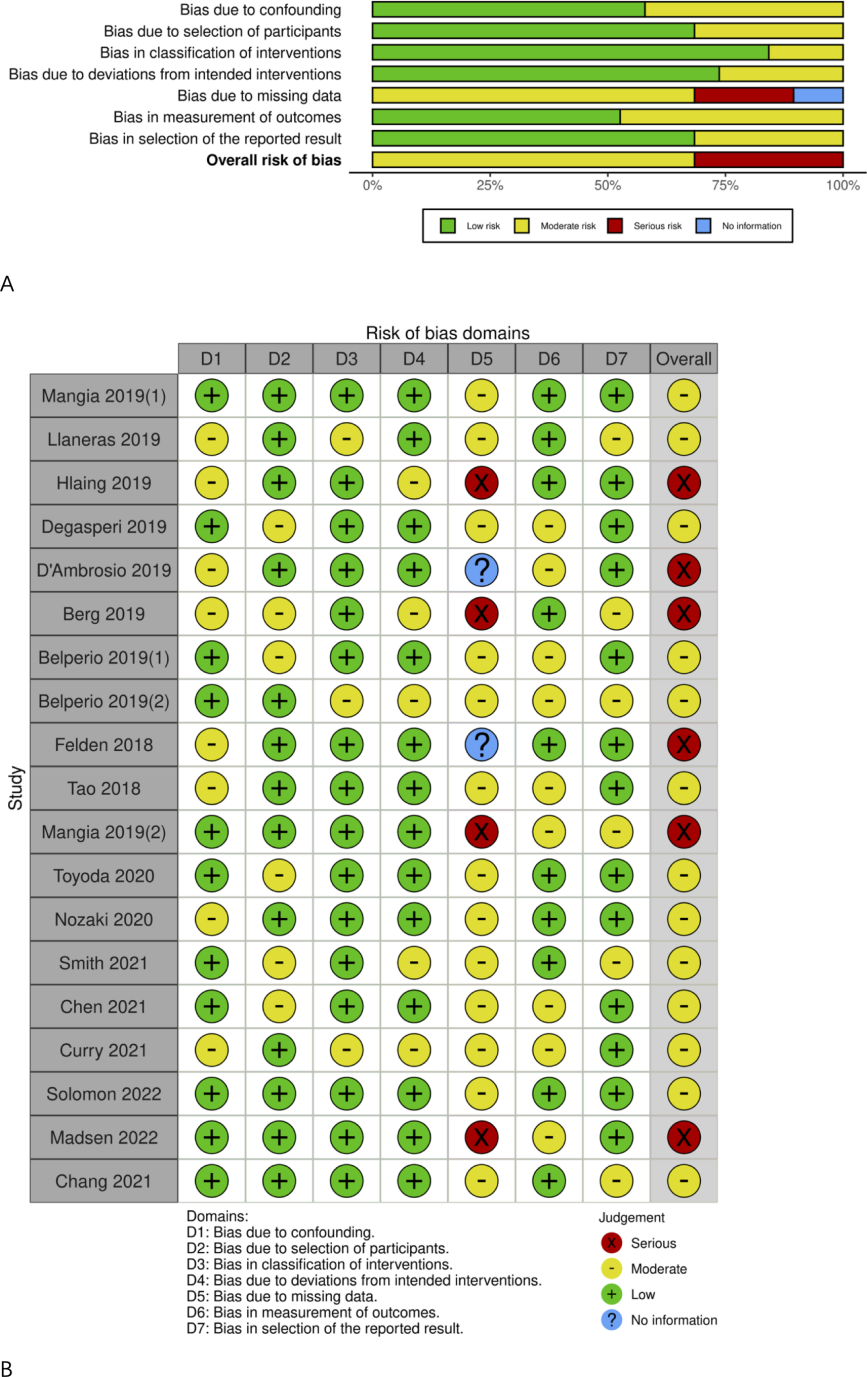
Risk of bias graph. **(A)** Risk of bias item among studies; **(B)** Risk of bias item in each study.

Detailed data on all genotypes instead of genotype 3 HCV patients in the majority of included articles were available, as shown in **Table 1**. The risk of bias due to missing data was moderate or high, as presented in **Figure 5**. The Egger’s test showed no significant publication bias (*t*=0.51, DF=17, P=0.6150).

## Discussion

With the approval of the direct-acting antivirals, the landscape of HCV treatment has significantly changed since 2015. New regimens and their combinations have been researched to resolve difficulties through antiviral therapy.

The pooled SVR12 rate of 3,177 HCV-GT3 patients who received SOF+VEL ± RBV, GLE+PIB, and SOF+VEL+VOX was 94.00% in the meta-analysis of rates in real-world settings. Patients with decompensated cirrhosis, prior DAA treatment-failure, HBV/HCV or HCV/HIV coinfection, chronic kidney disease, HCC, or a prior liver transplant who were considered difficult to treat were involved. The SVR12 rate was 90.50% (n=694/777) and 95.70% (n=1508/1617) in the patients with and without cirrhosis, respectively, and 88.41% (n=430/502) and 96.79% (n=2055/2201) in the treatment-experienced and treatment-naïve patients, respectively. A decrease of approximately 5.20% in the SVR rate in the patients with cirrhosis and an 8.38% decrease in the treatment-experienced patients were observed. Thus, the fibrosis stage and history of antiviral treatment might significantly impact the antiviral effectiveness. Furthermore, the fibrosis stage and particularly treatment history may significantly affect the SVR rates.

In the ASTRAL-3 study reported by Foster et al. (37), the SVR12 rate was 97% (n=191/197) and 91% (n=73/80) in HCV GT3 patients without cirrhosis and with compensated cirrhosis, respectively, and 97% (n=200/206) and 90% (n=64/71) in treatment-naïve and treatment-experienced patients, respectively. A retrospective study on patients who had compensated cirrhosis or advanced fibrosis had an SVR12 rate of 95% (n=145/153) in GT3 patients (38). Through subgroup analysis, the SVR12 rate of the treatment-experienced patients prescribed SOF+VEL ± RBV was 90.29% (n=270/312), which was lower than that of the treatment-naive patients (96.40%, n=1914/2054). Similar decreases were observed in the subgroup populations that received SOF+VEL+VOX. The SVR12 rate of patients with cirrhosis treated with SOF+VEL ± RBV was 91.10% (n=647/723), which was lower than that of patients without cirrhosis (94.61%, n=1339/1439), as well as that those that received GLE+PIB and SOF+VEL+VOX.

High SVR rates of patients with GLE+PIB have been reported in registration trials in recent years, ranging between 95% and 100% (39). The effect of HCV genotype, fibrosis stage, history of antiviral treatment, HCC, and advanced chronic kidney disease (CKD) on the efficacy of GLE+PIB seemed to be limited because of the excellent SVR rate (30). In the present analysis, the SVR12 rate of GT3 patients treated with GLE+PIB was 96.96% (n=429/448) in real-world settings. When analyzing a subgroup of patients with cirrhosis and treatment experience, the SVR rate results did not fluctuate significantly, mirroring those reported in previous trials.

Belperio et al. reported that among 13 GT3 patients with prior SOF/VEL exposure, the SVR rates were 100% (n=6/6) in those without cirrhosis and 71.4% (n=5/7) in those with cirrhosis. Thus, they considered that cirrhosis occurring with prior SOF/VEL exposure may augment the risk of relapse rather than cirrhosis alone (25). All the GT3 patients enrolled in the four studies of this meta-analysis that received SOF+VEL+VOX were treatment-experienced with SOF/LDV, OBV/PTV-r+DSV, SOF/VEL, or SOF/DCV. The pooled SVR12 rate was 83.81% (n=149/179) in the patients with and without cirrhosis. The SVR rate of the patients with cirrhosis and prior DAA exposure was 82.84% (n=30/36) and 92.68% (n=36/39), respectively, compared to the patients with prior DAA exposure but without cirrhosis. A decrease of 9.84% showed that the GT3 patients with cirrhosis and prior DAA failure were a more difficult-to-treat cohort.

Previous studies indicated that genotype 3 HCV with variants such as A30K, L31M, and Y93H of NS5A was refractory (40–42). Zeuzem et al. (40) reported that in the ENDURANCE-3 trial, GT3 patients with the A30K mutation at baseline had a lower SVR12 rate. However, most patients achieved SVR regardless of the A30K variant. In the ASTRAL-3 study, an SVR rate of 84% in patients with Y93H substitution compared to that of 97% in patients without the substitution was attained from patients who received SOF/VEL (37). Sarrazin et al. (43) reported that SVR12 rates were similar in patients with/without NS3 and/or NS5A resistance-associated variants (RASs) and patients with/without VOX- or VEL-specific RASs who received SOF + VEL + VOX for 12 weeks. Seven articles in this meta-analysis (19, 21, 22, 25, 26, 30, 31) completed the RAVs test, concluding that RASs may not be associated with a lower SVR rate. Nozaki et al. (30) indicated that the effect of RASs on therapeutic results was limited because of the 99.1% overall SVR12 rate. It may not be necessary to test for RASs before treatment because of the high SVR rates in patients completing therapy.

The limitations of this meta-analysis included high heterogeneity in the baseline characteristics and clinical features of the patients, along with a small number of patients who received SOF+VEL+VOX. More studies are needed in order to analyze the real-world antiviral effectiveness of DAAs in chronic HCV GT3-infected patients.

## Conclusions

In conclusion, SOF+VEL ± RBV, GLE+PIB, and SOF+VEL+VOX had good antiviral effectiveness for chronic HCV-GT3 infection in real-world settings. Factors such as cirrhosis and treatment experience, especially previous DAA treatment failure, may influence the SVR rate.

## Data Availability

The raw data supporting the conclusions of this article will be made available by the authors, without undue reservation.
